# Real-life assessment of aripiprazole long-acting injectable combined with brexpiprazole in schizophrenia: a naturalistic non-interventional prospective follow-up case series from Hong Kong

**DOI:** 10.1192/j.eurpsy.2026.12180

**Published:** 2026-07-31

**Authors:** A. K. K. Chung, C. Y. Tse, K. C. Law

**Affiliations:** Department of Psychiatry, University of Hong Kong, Hong Kong, Hong Kong

## Abstract

**Introduction:**

Antipsychotics are the mainstay of treatment for schizophrenia spectrum disorders. In Hong Kong, 41% of the practicing psychiatrists preferred to switch or add a long-acting injectable (LAI) antipsychotics to address non-adherence in patients with schizophrenia. Currently, there is no recommendation on next-step treatment strategy if the patients are having incomplete remission or acute exacerbations of schizophrenia whilst receiving aripiprazole LAI at its recommended (maximum) dose. Adding another SGAs to this special cohort appears to be rational. Brexpiprazole, a serotonin-dopamine activity modulator, might be an ideal choice as it retains the benefits of aripiprazole from its domapine system stabilizing property. Here, we reported a case series of patients with schizophrenia prescribed with the combination therapy of aripiprazole LAI and brexpiprazole on its efficacy and tolerability in a real-life clinical setting.

**Objectives:**

The primary objective of this study was to assess the efficacy measured by Brief Psyhiatric Rating Scale (BPRS) and Clinical Gobal Impression (CGI) in patients receiving combination therapy with aripiprazole LAI and brexpiprazole after 6 months. The secondary objective was to assess its tolerability using the Simpson-Angus Scale (SAS) and Barnes Akathisia Rating Scale (BARS).

**Methods:**

Adult patients diagnosed with schizophrenia and were receiving the combination therapy with aripiprazole LAI and brexpriprazole between January 2022 to June 2023 were prosepctively follow-up for 6 months in a local psychiatry specialist out-patient clinic. The efficacy and tolerability of this combination therapy were assessed at baseline, 3rd and 6th month. The pre- and post- treatemnt effects were analyzed using linear mixed model with time as a fixed effect.

**Results:**

Five patients (3 males and 2 females) participated. Their mean age was 38.4 years (range: 25 - 48 years).The mean number of antipsychotics received prior to the combination therapy was 4 (range: 2 - 7), suggesting a treatment-resistant course for their schizophrenia. One patient (25 years old female) withdrew her consent after 3 months due to muscle rigidity. The mean dosages of the combination therapy for aripiprazole LAI and brexiprazole were 400mg and 1.9mg at 3 months, and 400mg and 2.375mg at 6 months, respectively. Across all assessment scales, no significant efficacy measured by BPRS and CGI were observed, and no significant intolerability measured by SAS and BARS were noted (all ps >0.05) .

**Conclusions:**

The results from this case series suggested that patients receiving the combination therapy of aripiprazole LAI and brexpiprazole did not show significant improvements in the schizophrenia symptom severity across the 6-month period. Nevertheless, this combination are in general well tolearated.

**Disclosure of Interest:**

None Declared

